# Increased clusterin levels after myocardial infarction is due to a defect in protein degradation systems activity

**DOI:** 10.1038/s41419-019-1857-x

**Published:** 2019-08-13

**Authors:** Annie Turkieh, Sina Porouchani, Olivia Beseme, Maggy Chwastyniak, Philippe Amouyel, Nicolas Lamblin, Jean-Luc Balligand, Christophe Bauters, Florence Pinet

**Affiliations:** 1 Inserm, University of Lille, CHU Lille, Institut Pasteur de Lille, U1167—RID-AGE—Facteurs de Risque et Déterminants Moléculaires des Maladies Liées au Vieillissement, F-59000 Lille, France; 2 Fédération Hospitalière Universitaire (FHU), REMOD-VHF, Lille, France; 3 Institut de Recherche Experimentale et Clinique, Pole of Pharmacology and Therapeutics and Cliniques Universitaires Saint-Luc, Université Catholique de Louvain, Brussels, Belgium

**Keywords:** Biochemistry, Medical research

## Abstract

Clusterin (CLU) is induced in many organs after tissue injury or remodeling. Recently, we show that CLU levels are increased in plasma and left ventricle (LV) after MI, however, the mechanisms involved are not yet elucidated. On the other hand, it has been shown that the activity of the protein degradation systems (PDS) is affected after MI with a decrease in ubiquitin proteasome system (UPS) and an increase in macroautophagy. The aim of this study was to decipher if the increased CLU levels after MI are in part due to the alteration of PDS activity. Rat neonate cardiomyocytes (NCM) were treated with different modulators of UPS and macroautophagy in order to decipher their role in CLU expression, secretion, and degradation. We observed that inhibition of UPS activity in NCM increased CLU mRNA levels, its intracellular protein levels (p-CLU and m-CLU) and its secreted form (s-CLU). Macroautophagy was also induced after MG132 treatment but is not active. The inhibition of macroautophagy induction in MG132-treated NCM increased CLU mRNA and m-CLU levels, but not s-CLU compared to NCM only treated by MG132. We also demonstrate that CLU can be degraded in NCM through proteasome and lysosome by a macroautophagy independent pathway. In another hand, CLU silencing in NCM has no effect either on macroautophagy or apoptosis induced by MG132. However, the overexpression of CLU secreted isoform in H9c2 cells, but not in NCM decreased apoptosis after MG132 treatment. Finally, we observed that increased CLU levels in hypertrophied NCM and in failing human hearts are associated with proteasome inhibition and macroautophagy alteration. All these data suggest that increased CLU expression and secretion after MI is, in part, due to a defect of UPS and macroautophagy activities in the heart and may have a protective effect by decreasing apoptosis induced by proteasome inhibition.

## Introduction

Clusterin (CLU) is a protein constitutively expressed in almost mammalian tissues and is highly conserved across species. Its predominant form is a secreted heterodimeric glycoprotein (s-CLU, 75–80 kDa) obtained by the proteolytic cleavage of its precursor form (p-CLU, ~60 kDa) to generate alpha and beta chains of ~40 kDa that are glycosylated and assembled to give the mature form (m-CLU) that will be then secreted in physiological fluids^1^. Other CLU species are detected in some stress conditions and are found in different cellular compartments (mitochondria, cytoplasm, and nucleus). The role of CLU is complex depending on the CLU isoforms, localization, and cellular types. Several functions have been proposed such as lipid transport, apoptosis regulation protein degradation, and epithelial-to-mesenchymal transition induction^1–3^. CLU is induced in many organs where tissue injury or remodeling occurs. Several studies have shown increased CLU levels in the heart and plasma at early stage after myocardial infarction (MI)^3–5^. Recently, we showed that CLU levels are increased in plasma of patients at late stage after MI and are associated with left ventricle remodeling (LVR)^6^. We also demonstrated that CLU is increased in LV of post-MI rats and in LV of heart failure (HF) patients. Furthermore, CLU expression in plasma and LV was depending on the stage after MI suggesting that different mechanisms are involved in CLU regulation in HF post-MI^6^.

On the other hand, it has been shown that the activity of the protein degradation systems (PDS) is affected after MI with a decrease of ubiquitin proteasome system (UPS)^7,8^ and an increase of macroautophagy^9^ activities. The UPS is the major pathway for protein turnover and degradation of damaged or misfolded proteins in most organs including the heart. It consists in the ubiquitination of target proteins and their degradation by the proteasome^10^. The macroautophagy is an important proteolytic mechanism that regulates the homeostasis of long-lived proteins, macromolecules and cell organelles by the formation of an autophagosome which will then be fused with the lysosome to degrade its contents^10^. Despite that UPS and macroautophagy are different mechanisms, a crosstalk between these two pathways in protein quality control was observed in several cells including cardiac cells^11–17^. Activity alteration of these two systems was observed in some heart diseases and seems to be involved in cardiac remodeling and dysfunction^18^. It was shown that proteasome activity is decreased during MI and in HF patients with dilated cardiomyopathy and is involved in cardiac dysfunction^19–21^. Macroautophagy is induced after MI but its activity and its effect varied at different stages after MI. It was shown that macroautophagy inhibition is protective during reperfusion^22^; however, its induction in the late stage post-MI decreased LVR and improved cardiac function^9,23,24^. Furthermore, it was shown in noncardiac cells, an increase of CLU expression under stress conditions resulting in proteasome inhibition and/or macroautophagy induction^25,26^ and that macroautophagy can also be regulated by CLU^26,27^. However, no data on the relationship between CLU and these two systems have been yet studied in cardiac cells.

Our aims are (1) to verify whether the increase of CLU plasma and cardiac levels following MI could be, in part, a consequence of the alteration of one or more of the PDS activity and (2) to verify if CLU can be associated to LVR by regulating these systems. For that purpose, we first studied the effect of proteasome and/or macroautophagy activity alteration on CLU expression, secretion and degradation in isolated rat neonate cardiomyocytes (NCM) and then studied the role of CLU on macroautophagy regulation and cellular apoptosis induced after proteasome inhibition in NCM. Finally, we validated the association between CLU expression and the alteration of PDS activity in an in vitro model of hypertrophied NCM, a mechanism observed during LVR, and in failing human hearts.

## Results

### CLU expression and secretion are increased after inhibition of proteasome and macroautophagy activities in cardiomyocytes

**Fig. 1:**
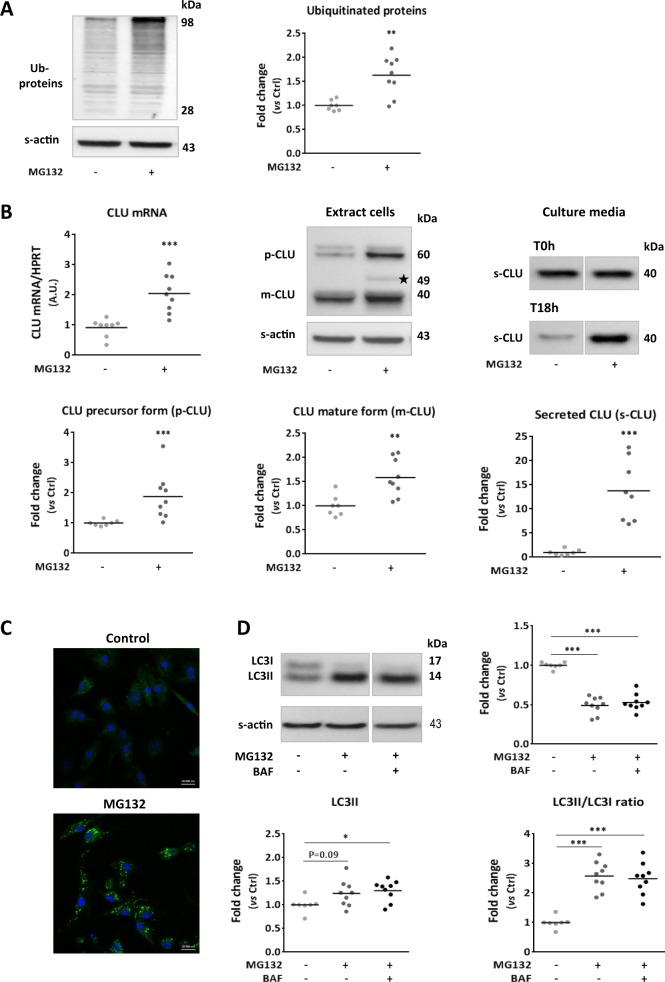
Inhibition of proteasome activity increased clusterin expression and secretion associated with macroautophagy induction in cardiomyocytes. **a** Representative western blots and quantification of ubiquitinated proteins levels in MG132-treated NCM (10 µmol/L, 18 h) (*n* = 9) compared to control cells (*n* = 7). **b** Analysis of clusterin (CLU) expression and secretion in NCM after MG132 treatment. Quantification by RT-qPCR, of CLU mRNA levels (upper left panel) in the MG132-treated cells (*n* = 9) compared to control cells (*n* = 8). Representative western blots (upper middle and right panels) and quantification (lower panels) of intracellular levels of precursor (p-CLU) and mature (m-CLU) forms of clusterin and its secreted form (s-CLU) in the MG132-treated cells (*n* = 9) compared to control cells (*n* = 7). * indicates the nuclear isoform of clusterin. The culture medium is collected from the same cells after 24 h serum-deprivation corresponding to the beginning of treatment (T0h) and after 18 h of treatment (T18h). The levels of s-CLU quantified at T0h are used as normalizer. **c** Autophagic vaccuoles were stained with cyto-ID^TM^ in control and MG132-treated NCM. DAPI stained nuclear DNA in blue. Scale bar: 20 µm. **d** Representative western blots and quantification of LC3 proteins levels and LC3II/LC3I ratio in NCM pre-treated or not with Bafilomycin (BAF, 50 nmol/L) 4 h before the end of MG132-treatment (*n* = 9) compared to control cells (*n* = 7). For qPCR analysis, HPRT was used to normalize CLU expression and the data are expressed in arbitary units (A.U.). For Western blot analysis, sarcomeric actin (S-actin) was used to normalize intracellular proteins levels. Data are expressed as individual and mean fold change in proteins levels relative to control cells. Statistical significance was determined by Wilcoxon–Mann Whitney test. *P < 0.05, **P < 0.01, ***P < 0.001 vs. control cells

We first studied the effect of PDS alterations on CLU expression and secretion in NCM treated with MG132 for 18 h in order to inhibit proteasome activity as verified by significant accumulation of ubiquitinated proteins (Fig. 1a). We showed a significant increase of CLU mRNA levels, its intracellular proteins levels (precursor (p-CLU) and mature forms (m-CLU)) and its secreted form (s-CLU) in the culture media after MG132 treatment compared to control cells (Fig. 1b).

We analyzed the impact of proteasome inhibition on macroautophagy induction in our model and observed, by cytoID staining, an increased number of autophagosomes (Fig. 1c), and a significant decrease of LC3I level, a trend increase of LC3II level and a significant increase of LC3II/LC3I ratio compared to control cells (Fig. 1d). To determine if macroautophagy is active, NCM were treated by bafilomycin (BAF) 4 h before the end of MG132 treatment. The inhibition of autophagosome–lysosome fusion has no effect on either LC3II level or LC3II/LC3I ratio (Fig. 1d) showing that macroautophagy is induced in cardiomyocytes after proteasome inhibition but it is not active.

**Fig. 2:**
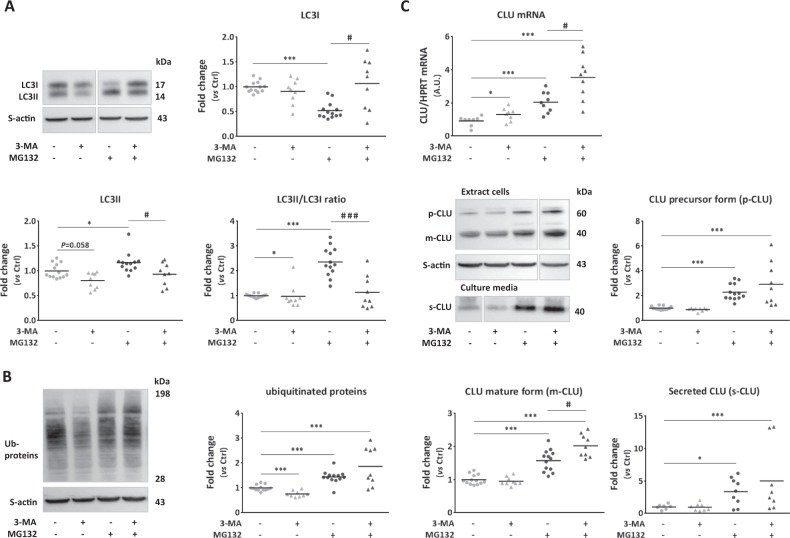
Inhibition of macroautophagy induction increased clusterin expression in MG132-treated cardiomyocytes. **a**, **b** Representative western blots and quantification of LC3 proteins levels, LC3II/LC3I ratio (**a**) and ubiquitinated proteins levels (**b**) in NCM treated with 3-methyladenine (3-MA, 10 mmol/L, 19 h) (*n* = 9) and/or MG132 (10 µmol/L, 18 h) (*n* = 9 and 13, respectively) compared to control cells (*n* = 13). **c** Analysis of clusterin (CLU) expression and secretion in NCM after the different treatments. Quantification by RT-qPCR, of CLU mRNA levels (upper panel) in the treated NCM (*n* = 8–9/group) compared to control cells (*n* = 8). Representative western blots and quantification of intracellular levels of precursor (p-CLU) and mature (m-CLU) forms of clusterin (*n* = 9 and 13/group, respectively) and its secreted form (s-CLU) (*n* = 8–9/group) in the treated NCM compared to control cells. For qPCR analysis, HPRT was used to normalize CLU expression and the data are expressed in arbitary units (A.U.). For western blot analysis, sarcomeric actin (S-actin) was used to normalize intracellular proteins levels and total mRNA levels were used to normalize secreted CLU levels. Data are expressed as individual and mean fold change in proteins levels relative to control cells. Statistical significance was determined by Wilcoxon–Mann Whitney test. *P < 0.05, ***P < 0.001 vs. control cells and ^#^*P* < 0.05, ***P < 0.001 vs. MG132-treated cells

To verify if increased CLU expression in MG132-treated cells is the consequence of proteasome inhibition or macroautophagy induction, NCM were treated by 3-MA to inhibit specifically macroautophagy. Macroautophagy inhibition was efficient alone or on MG132-treated NCM as shown by significant decreased LC3II level and significant decrease of LC3II/LC3I ratio (Fig. 2a). Interestingly, 3-MA alone induced proteasome activity as shown by the decreased ubiquitinated proteins level and the co-treatment induced a significant increase of the ubiquitinated proteins (Fig. 2b). 3-MA alone only increased CLU mRNA level without any impact on intracellular and secreted CLU levels compared to control cells (Fig. 2c). However, the co-treatment of NCM by 3-MA and MG132 increased CLU mRNA level and m-CLU level compared to MG132-treated cells and had the greatest effect on CLU expression compared to control cells (Fig. 2c). All these results show that CLU expression is increased when proteasome and macroautophagy are inactive.

### CLU is degraded by proteasome and lysosome with a macroautophagy independent pathway in cardiomyocytes

**Fig. 3:**
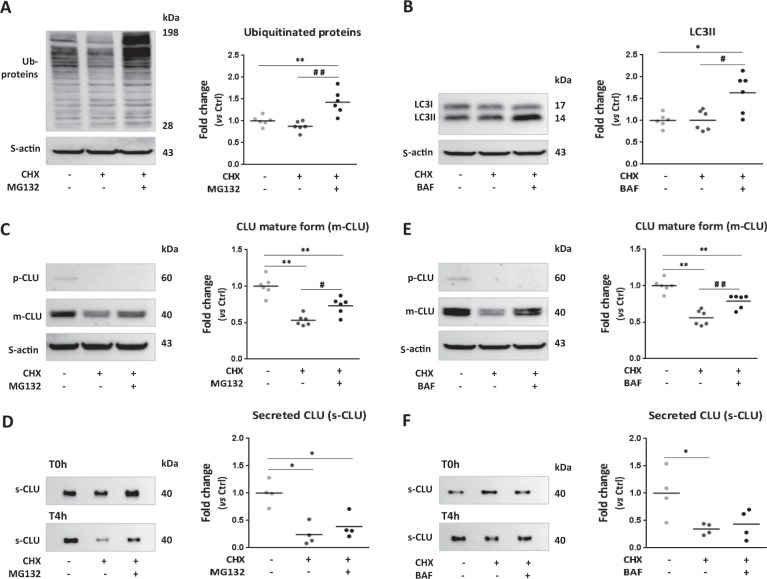
Clusterin is degraded by proteasome and lysosome in cardiomyocytes. **a** Representative western blots and quantification of ubiquitinated proteins levels in NCM treated with cycloheximide (CHX, inhibitor of novo protein synthesis, 100 μg/mL) in presence or absence of MG132 (10 µmol/L) for 4 h compared to control cells (*n* = 6/group). **b** Representative western blots of LC3 and quantification of LC3II proteins levels in CHX-treated NCM in presence or absence of BAF (50 nmol/L) for 4 h compared to control cells (*n* = 6/group). **c**–**e** Representative western blots of intracellular CLU proteins levels and quantification of CLU mature form (m-CLU) levels in CHX-treated NCM in presence or absence of MG132 (**c**) or BAF (**e**) for 4 h compared to control cells (*n* = 6/group). For all intracellular proteins levels, sarcomeric actin (S-actin) was used to normalization. **d**–**f** Representative western blots and quantification of secreted CLU proteins levels (s-CLU) in CHX-treated NCM in presence or absence of MG132 (**d**) or BAF (**e**) for 4 h compared to control cells (*n* = 4/group). The culture medium is collected from the same cells after 24 h serum-deprivation corresponding to the beginning of treatment (T0h) and after 4 h of treatment (T4h). The levels of s-CLU quantified at T0h are used as normalizer. Graph show individual and mean fold change in proteins levels relative to control cells. Statistical significance was determined by Wilcoxon–Mann Whitney test. *P < 0.05, **P < 0.01 vs. control cells and ^#^*P* < 0.05, ^##^*P* < 0.01 vs. CHX-treated cells

We hypothesized that the increased CLU proteins levels after proteasome and macroautophagy inactivation might be due, in part, to less degradation. For that purpose, we first identified which organelle, proteasome or lysosome might be involved in CLU degradation in cardiomyocyte. We treated the cells by cycloheximide (CHX) to inhibit de novo protein synthesis in the presence or absence of MG132 or BAF. CHX treatment alone had no effect on either the levels of ubiquitinated proteins (Fig. 3a) or LC3II levels (Fig. 3b) showing that the proteasome and the lysosome are active in CHX-treated NCM. As expected, we did not detect p-CLU in CHX-treated NCM showing that 4 h treatment is sufficient to inhibit de novo CLU synthesis in cardiomyocytes (Fig. 3c). Consequently, we observed a significant decrease of m-CLU and s-CLU levels after CHX-treatment (Fig. 3c, d). The co-treatment with MG132 inhibited proteasome activity as observed by the significant increased levels of ubiquitinated proteins (Fig. 3a) and of m-CLU levels compared to CHX-treated cells (Fig. 3c). However, we did not observe any effect on s-CLU levels (Fig. 3d) showing that accumulation of m-CLU after MG132 treatment is due to a decrease of its degradation by the proteasome and not to a decrease of its secretion. Furthermore, the co-treatment with BAF inhibited lysosome activity as observed by the accumulation of LC3II levels (Fig. 3b) and the significant increase of m-CLU levels (Fig. 3e) with no effect on s-CLU levels (Fig. 3f) compared to CHX-treated cells. These data suggest that both proteasome and lysosome activities are involved in CLU degradation in cardiomyocytes. To decipher the role of macroautophagy on CLU degradation in cardiomyocytes, we used an in vitro model of macroautophagy activation by “nutrients-deprivation” of NCM in Hank’s Balanced Salt Solution (HBSS) for 2 h. This model induced macroautophagy as shown by the significant increase of LC3II levels and LC3II/LC3I ratio (Supplementary Fig. 1a) without any effect on proteasome activity (Supplementary Fig. 1b). We did not observe any modulation of CLU mRNA (Supplementary Fig. 1c) nor intracellular CLU (Supplementary Fig. 1d) levels suggesting that macroautophagy is not involved in CLU degradation in cardiomyocytes.

### CLU is not involved on macroautophagy in MG132-treated cardiomyocytes

To evaluate the role of CLU on macroautophagy in cardiomyocyte, we silenced CLU by transfecting siRNA (siClu-1 and siClu-2) in NCM for 48 h before MG132 treatment. CLU silencing was efficient and specific as shown by the significant decrease of intracellular p-CLU and m-CLU levels (Supplementary Fig. 2). Indeed, CLU silencing had no effect on either LC3I or LC3II levels showing that CLU is not involved on macroautophagy in MG132-treated cells (Supplementary Fig. 2).

### CLU silencing and overexpression has no effect on apoptosis induced by MG132 in cardiomyocytes

**Fig. 4:**
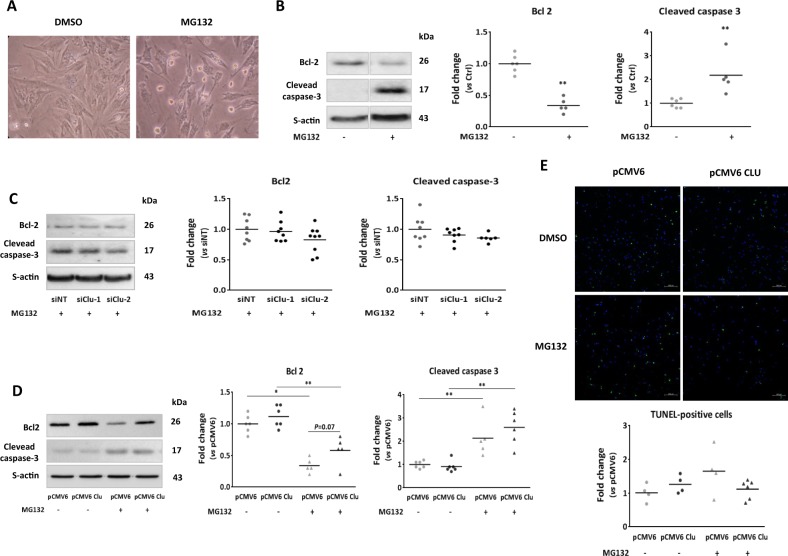
Clusterin silencing and overexpression had no effect on apoptosis induced by MG132 in cardiomyocytes. **a** Representative control- and MG132-treated NCM (10 µmol/L, 18 h) observed by phase-contrast microscopy. **b** Representative western blots and quantification of Bcl2 and cleaved caspase-3 proteins levels in MG132-treated NCM (*n* = 5) compared to control cells (*n* = 6). **c** NCM were transfected with siRNA non-target (si-NT) or siRNA targeting clusterin (siClu-1 and siClu-2) for 48 h before MG132 treatment (10 µmol/L, 18 h). Representative western blots and quantification of Bcl2 and cleaved caspase-3 proteins levels in MG132-treated NCM after CLU silencing (*n* = 8 and 6 for siClu-1 and siClu-2, respectively) compared to siNT-transfected NCM (*n* = 8). Sarcomeric actin (S-actin) was used to normalize proteins levels and data are expressed as individual and mean fold change in proteins levels relative to siNT-transfected cells. **d** NCM were transfected by empty vector (pCMV6) or vector overexpressing the secreted isoform of clusterin (pCMV6 Clu) for 48 h before MG132 treatment. Representative western blots and quantification of Bcl2 and cleaved caspase-3 proteins levels in transfected cells in presence and absence of MG132 (*n* = 6/group). Sarcomeric actin (S-actin) was used to normalize proteins levels and data are expressed as individual and mean fold change in proteins levels relative to pCMV6-transfected cells. **e** TUNEL staining (scale bar: 100 µm) in pCMV6 and pCMV6 Clu- transfected cells in presence or absence of MG132 and quantification of the percentage of TUNEL-positive cells in these 4 conditions normalized by the data obtained in pCMV6 group. (*n* = 9/group). Statistical significance was determined by Wilcoxon–Mann Whitney test. *P < 0.05, **P < 0.01 vs. nonstimulated condition

Previous studies showed an anti-apoptotic role of secreted CLU in different cell types^27–30^. In our model of cardiomyocyte, MG132 treatment increased NCM apoptosis as shown in phase-contrast microscopy (Fig. 4a) and by the significant decrease of Bcl2 levels and the significant increase of cleaved caspase-3 levels (Fig. 4b). To verify if increased CLU expression after MG132 treatment is protective for the cardiomyocyte, we first studied the effect of CLU silencing on apoptosis of NCMand we observed that CLU silencing had no effect on either Bcl2 or cleaved caspase-3 levels (Fig. 4c). Then, we overexpressed the secreted CLU isoform (pCMV6 Clu) in NCM (transient transfection) and in rat cardiomyoblasts H9c2 (stable transfection) in which CLU expression is very low for studying its effect on apoptosis.

In NCM, as previously observed, MG132 treatment increased apoptosis in pCMV6- and pCMV6 Clu-tranfected NCM (Fig. 4d) as observed by a significant decrease of Bcl2 levels, a significant increase of cleaved caspase 3 levels and a trend increase of transferase-mediated deoxyuridine triphosphate-biotin nick-end labeling (TUNEL)-positive cells number (Fig. 4d). Unexpectedly, the effect of MG132 treatment on CLU expression and secretion was only observed in pCMV6 Clu-transfected NCM (Supplementary Fig. 3). We observed a significant increase of p-CLU, m-CLU, s-CLU and a slight band at 49 kDa, as observed in Fig. 1b, that might correspond to the nuclear isoform (nCLU) of CLU that is expressed in some cells under stress conditions^31–33^. The overexpression of CLU in NCM (Supplementary Fig. 3) had no significant effect on MG132-induced apoptosis with a trend increase of Bcl2 and decrease of TUNEL-positive cells in pCMV6 Clu-transfected NCM after MG132-treatment compared to treated pCMV6-transfected cells (Fig. 4d).

In H9c2 cells, MG132 treatment was efficient as shown by the significant increased levels of ubiquitinated proteins (Supplementary Fig. 4a). This treatment also increased apoptosis as shown by the significant increase of cleaved caspase-3 levels (Supplementary Fig. 4b) associated with a significant increase of pCLU, m-CLU, and s-CLU in pCMV6 Clu-transfected H9c2 (Supplementary Fig. 4c). However, we did not observe the potential nCLU after MG132 treatment in this cell line. Interestingly, the overexpression of secreted CLU in H9c2 cells decreased the apoptosis induced by MG132 treatment as observed by the significant decrease of cleaved caspase-3 levels in treated pCMV6 Clu-transfected cells compared to treated pCMV6 (Supplementary Fig. 4b), showing a behavior different from NCM (Fig. 4d).

### Increased CLU expression in hypertrophied cardiomyocytes and human HF cardiac biopsies is associated with proteasome inhibition and macroautophagy alteration

In our recent study, we showed a significant increase of CLU expression and secretion in hypertrophied NCM (by treating the cells by isoproterenol) and a significant increase of CLU intracellular levels (p-CLU and m-CLU) in the human failing heart patients compared to non-failing hearts^6^. To verify if CLU expression is associated with PDS alterations in pathological conditions, we quantified the intracellular ubiquitinated proteins and LC3 levels in these samples.

**Fig. 5:**
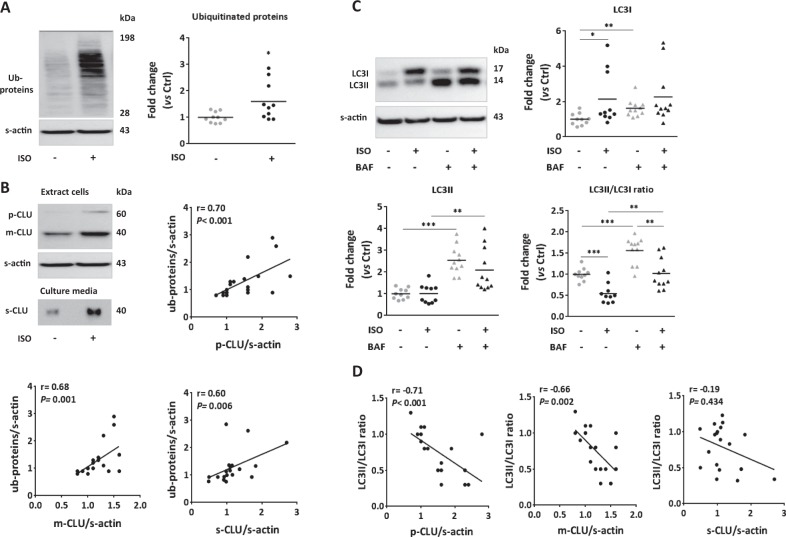
Increased clusterin expression in hypertrophied cardiomyocytes is associated with proteasome and macroautophagy inhibition. **a** Representative western blots and quantification of ubiquitinated proteins levels in NCM treated by isoproterenol (ISO, 10 µmol/L, 72 h) compared to control cells (*n* = 10/group). **b** Representative western blots of intracellular CLU proteins levels (precursor and mature forms) and its secreted form (top left panel) in NCM treated or not with ISO. Correlation of CLU precursor (top right panel), mature (bottom left panel) and secreted forms (bottom right panel) with ubiquitinated proteins levels in these cells. **c** Representative western blots and quantification of LC3 proteins levels and LC3II/LC3I ratio in ISO-treated NCM compared to control cells (*n* = 11/group). **d** Correlation of CLU precursor (left panel), mature (middle panel), and secreted form (right panel) with LC3II/LC3I ratio in NCM treated or not with ISO. For western blot analysis, sarcomeric actin (S-actin) was used to normalize proteins levels and data are expressed as individual and mean fold change in proteins levels relative to control cells. Statistical significance was determined by Wilcoxon–Mann Whitney or Spearman correlation test. *P < 0.05, **P < 0.01, ***P < 0.001 vs. nonstimulated condition or indicated values

In the “in vitro” model of cardiac hypertrophy, we showed a significant increase of ubiquitinated proteins levels in ISO-treated NCM (Fig. 5a), significantly positively correlated with p-CLU, m-CLU, and s-CLU levels (Fig. 5b). We also found a significant increase of LC3I levels and a significant decrease of LC3II/LC3I ratio, in presence and absence of bafilomycin on ISO-treated NCM showing that macroautophagy is inhibited in hypertrophied cardiomyocytes (Fig. 5c). Furthermore, we showed that LC3II/LC3I ratio is significantly negatively correlated with p-CLU and m-CLU in these cells but not with s-CLU (Fig. 5d).

**Fig. 6:**
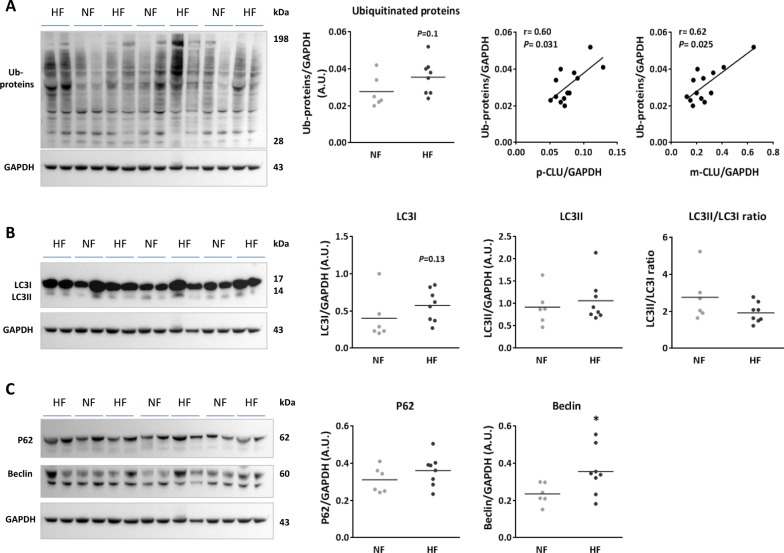
Increased Clusterin expression in human cardiac biopsies from control and heart failure patients is associated with proteasome and macroautophagy alteration. **a** Western Blot and quantification (left panels) of ubiquitinated proteins levels in LV biopsies obtained from human nonfailing controls (NF, *n* = 6) and heart failure patients (HF, *n* = 8). Correlation of CLU precursor and mature form with ubiquitinated proteins levels in these samples (right panels). **b**, **c** Analysis of macroautophagy in HF. Western Blot and quantification of LC3 proteins levels, LC3II/LC3I ratio (**b**), p62 and Beclin-1 proteins levels (**c**) in LV biopsies obtained from HF compared to NF patients. GAPDH was used to normalize all proteins levels and data are expressed as individual and mean fold change in proteins levels relative to NF patients. Statistical significance was determined by Wilcoxon–Mann Whitney or Spearman correlation test. *P < 0.05 vs. NF

In cardiac human biopsies, we showed a trend of increased levels of ubiquitinated proteins in failing hearts (HF) compared to nonfailing (NF) hearts, significantly positively correlated with p-CLU and m-CLU levels (Fig. 6a). We also showed a trend of increased levels of LC3I without any difference on LC3II levels or LC3II/LC3I ratio in HF patients compared to NF patients (Fig. 6b). Because we are unable to study the autophagic flux in these samples, we quantified P62 and beclin-1, other markers of macroautophagy induction and activation and we showed a trend of increase of P62 levels and a significant increase of beclin-1 levels in HF patients compared to NF patients (Fig. 6c) suggesting that macroautophagy is induced but not active in HF patients.

All of these data show that increased CLU expression in hypertrophied NCM and in LV of HF patients is associated with proteasome inhibition and macroautophagy alteration.

## Discussion

Protein quality control plays an essential role in maintaining protein homeostasis in cardiomyocytes^10^. It detects and repairs misfolded/unfolded proteins and if the repair fails, the abnormal proteins are degraded by the proteasome or lysosome. UPS and autophagy are the two main pathways responsible for clearance of defective proteins and alteration of their activities has been described in some heart diseases and might be involved in cardiac remodeling^18,34,35^. Several studies have concluded that proteasome activity is inhibited during LVR and HF post-MI, although macroautophagy is induced but its activity is not sufficient to remove all accumulated proteins resulting in increased oxidative stress and cell apoptosis^7–9,21,23,24^. Recently we showed that CLU is increased in LV and plasma after MI^6^, however, the mechanisms involved in this increase are unknown. In the other hand, it was shown, in noncardiac cells, increased CLU expression under stress conditions resulting in proteasome inhibition and/or macroautophagy induction^25,26^. However, no data on the relationship between CLU and these two systems have been yet studied in cardiac cells. Our aim was therefore to verify whether the increased CLU plasma and cardiac levels following MI could be, in part, a consequence of the alteration of the PDS activity by studying the effect of proteasome and macroautophagy alterations in cardiomyocytes.

We found that inhibition of proteasome activity in cardiomyocytesincreased CLU expression at mRNA and intracellular proteins levels. This increase of CLU expression might be explained by the binding of two transcription factors, heat-shock factor 1 and 2, to a particular heat-shock element present in CLU promoter named CLU element, as previously shown in human cancer cells^25,36^. Interestingly, we found increased s-CLU in culture media of MG132-treated NCM, as shown in cancer cells^37^, suggesting that increased intracellular CLU levels in cardiac cells is not due to a decrease of its secretion. However, the inhibition of proteasome activity partially restores m-CLU levels in NCM treated by an inhibitor of de novo protein synthesis suggesting that the increased intracellular CLU levels might be due, in part, to less degradation by the proteasome. This result is in agreement with other studies showing that CLU can be ubiquitinylated and degraded by proteasome in cancer cells^25,31^. All these data suggest that proteasome inhibition in cardiomyocyte increases CLU expression and its secretion by decreasing its degradation.

The UPS and autophagy are two complementary proteolytic pathways required for maintaining intracellular proteostasis, with a crosstalk in different cells including cardiac cells^17^. It was shown that inhibition of proteasome activity induced macroautophagy^11–14^, however, the effect of macroautophagy inhibition on UPS function showed contradictory results^12,15,16^. In our model, UPS inhibition induced only inactive macroautophagy. To decipher the role of macroautophagy on CLU regulation upon UPS inhibition in NCM, macroautophagy induction was inhibited by 3-MA treatment in presence and absence of MG132. We observed that 3-MA alone increased CLU mRNA levels without any effect on CLU intracellular proteins levels and increased proteasome activity, suggesting that CLU is partially degraded by proteasome, explaining why intracellular CLU levels are unchanged. This hypothesis was confirmed by the increased CLU mRNA and m-CLU levels in the NCM co-treated by 3-MA and MG132. All these data suggest that CLU in NCM is increased upon inhibition of both proteasome and macroautophagy.

Several studies showed that CLU plays a protective role by regulating protein homeostasis both at extracellular^38,39^ and intracellular compartments^26,27,40,41^ under stress conditions. For example, in Alzheimer disease, secreted CLU binds to extracellular Ab1–42 aggregates and allows their degradation by the lysosome^42^. It was also shown that CLU can regulate macroautophagy in cancer cells by inducing LC3 lipidation^26^. We hypothesized that CLU is increased after the inhibition of PDS in order to eliminate the damaged and aggregated proteins. Our CLU silencing experiments, in addition to other unshown data, allow us to conclude that CLU is not involved in macroautophagy regulation in cardiomyocytes.

An antiapoptotic role of CLU has been described in some stress conditions^28–30^, but the exact role of CLU on cellular survival is difficult to assess due to diverse and even opposite role of intracellular CLU isoforms in addition to the predominant secreted isoform in damaged cells^33,43^. In our model, we observed that UPS inhibition induced cell apoptosis associated with increased CLU levels; however, the silencing of CLU or its overexpression in MG132-treated NCM has no effect. This result can be explained by the nCLU isoform described to be proapoptotic (49 kDa), in addition to the anti-apoptotic secreted isoform in the same cells as in cancer prostate cells after MG132 treatment^31^. This hypothesis was validated by overexpression of s-CLU in H9c2 cells which induced a decrease of cell apoptosis upon UPS inhibition associated with absence of nCLU. These data suggest a protective role of s-CLU iafter proteasome inhibition in cardiomyocyte.

In our recent study^6^, we showed that CLU expression and secretion are increased in hypertrophied NCM, a mechanism observed during LVR post-MI and in cardiac biopsies obtained from HF patients. Here, we found that proteasome and macroautophagy activities are altered in in vitro NCM cultures and cardiac biopsies. Interestingly, CLU intracellular and secreted levels were positively correlated to the levels of ubiquitinated proteins. These results validate the association between CLU expression and the alteration of PDS activities under cardiac pathological conditions and suggest that the increased CLU expression after MI can be due, in part, to a decrease of proteasome and macroautophagy activities.

To conclude, this is the first study on the effect of PDS alteration on CLU regulation in cardiomyocytes. Proteasome inhibition increased CLU expression and secretion and decreased its degradation in NCM. We also validated it in hypertrophied NCM and in human cardiac biopsies suggesting that increased CLU during HF post-MI could be due in part to alteration of PDS activities. The inhibition of both proteasome and macroautophagy activities is associated with apoptosis of cardiac cells. As damaged proteins are accumulated after PDS alterations, the protective effect of CLU could be due to its capacity to regulate proteins degradation. It will be important to verify if CLU, by its chaperone activity, could bind proteins aggregates and degrade them through the lysosome or if CLU may regulate or be involved in chaperone-mediated autophagy mechanism in cardiomyocytes.

## Materials and methods

### Human heart biopsies

Tissues from HF and NF human hearts were obtained respectively from Lille University Hospital (France) and from Catholic University of Leuven (Belgium). Explanted heart tissues were obtained from patients undergoing heart transplantation for end-stage ischemic HF and from patients died of noncardiac causes. Samples were quick-frozen and stored at −80 °C. All materials from patients and controls were recovered as surgical waste with informed consent of the donors and with approval of the local ethical boards and according to the Declaration of Helsinki.

### Cellular models

#### Primary cultures of neonate rat cardiomyocytes (NCM)

Primary cultures of rat neonatal contractile cardiac myocytes (NCMs) were prepared from heart ventricles of 1- or 2-day-old rats, killed by decapitation, minced in a balanced salt solution containing 20 mmol/L HEPES, 120 mmol/L NaCl, 1 mmol/L NaH_2_PO_4_, 5.5 mmol/L glucose, 5.4 mmol/L KCl, and 0.8 mmol/L MgSO_4_ [pH 7.4] and then digested by using pancreatin and collagenase type II as previously described^6^. NCMs were purified by centrifugation of the cells at 2800 rpm for 30 min on Percoll (P4937, Sigma-Aldrich) gradients (58.5% and 40.5% in the balanced salt solution). NCMs were seeded at a density 6.5 × 10^5^ cells/well in 6-well plates coated with 10% of collagen (C8919, Sigma-Aldrich) and cultured in a medium containing 4 parts of Dulbecco’s modified Eagle’s medium (DMEM, D1152, Sigma-Aldrich) and 1 part of Medium199 (M199, M2520, Sigma-Aldrich), 10% fetal bovine serum (FBS, 30-2020, ATCC) and 1% penicillin and streptomycin (P/S, 10,000 U/mL) (15140-122, Invitrogen™, Life Technologies) at 37 °C under 5% CO_2_ atmosphere.

#### Cell treatment to inhibit and/or activate UPS and macroautophagy

After 48 h of seeding, cells were serum-deprived for 24 h and then treated with MG132 (M7449, Sigma-Aldrich) at 10 µM for 18 h^31^ to inhibit proteasome activity; or/and with 3-methyladenine (3-MA, M9281, Sigma-Aldrich) at 10 mM for 19 h to inhibit macroautophagy induction^44^. To study the autophagic flux, cells were treated with Bafilomycin (BAF, B1793, Sigma-Aldrich) at 50 nmol/L 4 h before the end of the treatment^45^. To study the CLU degradation by proteasome or lysosome, cells were treated with CHX (J66004, Alfa Aesar) at 100 µg/mL for 4 h^31^, to inhibit novo protein synthesis, in presence or absence of MG132 (10 µmol/L) or BAF (50 nmol/L), respectively. For all these treatments, cells cultured in serum-free medium with DMSO are used as control. To induce macroautophagy, cells were cultured in Hank’s Balanced Salt Solution (HBSS, 55037C, Sigma-Aldrich) for 2 h and compared to cells cultured in complete medium (with 10% stromal vascular fraction).

#### Hypertrophic treatment

After 24 h of seeding, cells were serum-deprived for 24 h and then treated with isoproterenol (10 μmol/L, Tocris Bioscience, 1747) for 72 h in the presence of 1% FBS. Cells cultured in medium with 1% FBS are used as control.

#### CLU silencing in cardiomyocytes

To inhibit the expression of CLU, NCM were transfected by 25 nmol/L of siRNA nontarget (NT) (Dharmacon, D-001810-10-20) as a control or siRNA- Clu 1 and 2 (Dharmacon, J-100279-09 and J-100279-10, respectivley) using Darmaphect according to the manufacturer’s instructions 48 h before MG132 treatment.

#### CLU overexpression in cardiomyocytes and H9c2 cells

Rat cardiac myoblasts (H9c2 cells, CRL-14146, ATCC) were cultured in Dulbecco’s Modified Eagle’s Medium (DMEM Glutamax, 31966, Gibco, Life Technologies) with addition of 10% (v/v) FBS and 1% P/S. Cells were cultured at 37 °C under 5% CO_2_ atmosphere.

To overexpress CLU, NCM and H9c2 cells were transfected with pCMV6 vector as a control or pCMV6- Clu vector (OriGene, RN214684) using Lipofectamine 2000 (Invitrogen) with a ratio 1:2 (DNA/Lipo) according to the manufacturer’s instructions. For stable transfection, cells were selected and cultured in appropriate media containing G418 (200 µg/mL) (A1720, Sigma-Aldrich).

### RNA extraction and quantitative real time-polymerase chain reaction (qRT-PCR)

RNA was extracted from NCM with QIAGEN RNeasy Mini Kit (Qiagen), as described by the manufacturers’s instructions. Then, 250 ng of RNA were reverse-transcribed using the miScript II RT kit (Qiagen). qRT-PCR was performed with the miScript SYBR Green PCR kit (Qiagen) on a Mx3005P Q-PCR system (Agilent Technologies), according to the manufacturer’s instructions. The sequences of the different primers (Eurogentec) used were: rat Clu (sense: GCTCCATAGCCCAGCTTTAC and antisense: ACTTCTCACACTGGCCCTTC), and rat HPRT (sense: ATGGGAGGCCATCACATTGT and antisense: ATGTAATCCAGCAGGTCAGCAA). The ΔΔCT method was used for data analysis.

### Western blot analysis

#### Protein extraction

H9c2 and NCM protein extracts were collected by scraping cells into ice-cold RIPA buffer as previously described^6^. Proteins from culture medium were precipitated with acetone (ratio 1:4) at −20 °C overnight and then resuspended in RIPA buffer as previously described^6^. Protein concentrations were determined with a Bradford-based method protein assay (Biorad, 500-0006).

#### Western blot

Proteins (20 µg of cellular protein extract and 5 µL of concentrated culture medium) were separated on 10% SDS-PAGE, transferred on 0.22 µm nitrocellulose membranes (Trans-Blot® Turbo^TM^ Transfert Pack, Bio-rad) and then incubated with antibodies as previously described^6^. The primary antibodies used for western blot analysis were: CLU (sc-6419), Bcl2 (sc-493), and GAPDH (sc-365062) antibodies from Santa Cruz Biotechnology; beclin-1 (#3738), LC3B (#2775), and cleaved caspase 3 (#9664) antibodies from Cell signaling; mono-and poly-ubiquitin antibody (BML-PW8810) from Enzo Life Sciences; P62 (610498) from BD Transduction Laboratories; β-actin antibody (A5316) from Sigma-Aldrich and sarcomeric-actin antibody (m0874) from Dako. The horseradish peroxidase-labeled secondary antibodies used were: anti-rabbit IgG (NA934V) and anti-mouse IgG (NA931) antibodies from GE healthcare and anti-goat IgG antibody (sc-2020) from Santa Cruz Biotechnology. The dilution of antibodies used for Western blot is provided for each sample analyzed (Supplementary Table 1). The Chemidoc® camera (Biorad) was used for imaging the membranes and densitometric measurements of the bands were analyzed with the Image Lab software (Bio-Rad).

### Autophagic vacuole staining

Autophagic vacuoles were detected by fluorescence microscopy using the Cyto-ID^™^ autophagy detection kit (Enzo Life Sciences). NCM were plated at a density of 8 × 10^8^ cells on coverslip coated with collagen in a 6-wells culture plate. After MG132 treatment, the cells were incubated with serum-free medium containing Hoechst (1/1000) and cyto-ID (1/500) for 30 min at 37 °C. The cells were then washed with the 1× Assay buffer and then fixed with 4% paraformaldehyde (43368, Thermo Fisher) for 20 min at room temperature. After 3 washes for 5 min with 1× Assay buffer, the coverslips were mounted on slides with glycerol 90%. Staining was visualized with the ×40 objectives of LSM710 confocal microscope followed by Zen image acquisition.

### Evaluation of apoptosis by TUNEL assay

Apoptosis was assessed by TUNEL (Roche) assay. NCM were plated at a density of 8 × 10^8^ cells on coverslip coated with collagen in 6-well plates. After MG132 treatment, cells were fixed with a fresh solution of 4% paraformaldehyde for 20 min and then permeabilized in 0.1% Triton X-100 for 20 min at room temperature. The cells were incubated with 50 μl TUNEL reaction mixture for 1 h at 37 °C in a humidified and dark atmosphere and then with DAPI for 10 min to stain nuclei. Three washes with phosphate-buffered saline 1× were performed after each step. The coverslips were then mounted on slides with glycerol 90%. Staining was visualized with the ×10 objective of LSM710 confocal microscope followed by Zen image acquisition.

### Statistical analysis

Data were analyzed with GraphPad Prism version 6.01 (GraphPad Software, San Diego, CA). Comparisons were made by Wilcoxon–Mann–Whitney test. Results were considered statistically significant if the *p* < 0.05. Correlations were carried out by Spearman correlation test.

## Supplementary information

Supplemental data

Supplemental table 1

Supplemental figure 1

Supplemental figure 2

Supplemental figure 3

Supplemental figure 4
