# Supplementary material for: Increased clusterin levels after myocardial infarction is due to a defect in protein degradation systems activity
Source: Cell Death Dis. 2019 Aug 13;10(8):608. doi: 10.1038/s41419-019-1857-x (PMC6691115; doi:10.1038/s41419-019-1857-x)
Supplement: Supplementary file 1 — Supplemental data [file 41419_2019_1857_MOESM1_ESM.docx]

**SUPPLEMENTAL DATA**

**Increased clusterin levels after myocardial infarction is due to a defect in protein degradation systems activity**

Annie Turkieh^1,2^, Sina Porouchani^1,2^, Olivia Beseme^1^, Maggy Chwastyniak^1^, Philippe Amouyel^1^, Nicolas Lamblin^1,2^, Jean-Luc Balligand^3^, Christophe Bauters^1,2^, Florence Pinet,^1,2^

**Supplemental table and figures legends**

**Suppl. Table 1: List and dilution of antibodies used for western blot analysis**

**Suppl. Figure 1: Macroautophagy is not involved in Clusterin degradation in cardiomyocytes.** The isolated rat neonatal cardiomyocytes (NCM) cultured in Hank's Balanced Salt Solution (HBSS) for 2h to induce macroautophagy were compared to cells cultured in complete medium (with 10% SVF). **A-B:** Representative western blots and quantification of LC3 proteins levels, LC3II/LC3I ratio (**A**) and ubiquitinated proteins levels (**B**) in HBSS-treated NCM (n=7) compared to control cells (n=9). **C:** Quantification by RT-qPCR, of CLU mRNA levels in HBSS-treated NCM compared to control cells (n=9/group). **D:** Representative western blots and quantification of intracellular levels of precursor (p-CLU) and mature (m-CLU) forms of clusterin in HBSS-treated NCM (n=7) compared to control cells (n=9). For qPCR analysis, HPRT was used to normalize CLU expression and the data are expressed in arbitary units (A.U.). For Western blot analysis, Sarcomeric actin (S-actin) was used to normalize intracellular proteins levels. Data are expressed as individual and mean fold change in proteins levels relative to control cells. Statistical significance was determined by Wilcoxon-Mann Whitney test. ***P*<0.01, ****P*<0.001 *vs* control cells.

**Suppl. Figure 2: Clusterin is not involved in macroautophagy regulation in cardiomyocytes.** The isolated rat neonatal cardiomyocytes (NCM) were transfected with siRNA non-target (si-NT) or siRNA targeting Clusterin (siClu-1 and siClu-2) for 48h before MG132 treatment (10 µM, 18h). Representative western blots (**A**) and quantification (**B**) of intracellular precursor (p-CLU) and mature (m-CLU) forms of Clusterin, LC3 proteins levels and LC3II/LC3I ratio in NCM after CLU silencing (n=7 and 6 for siClu-1 and siClu-2, respectively) compared to siNT-transfected cells (n=7). Sarcomeric actin (S-actin) was used to normalize proteins levels. Data are expressed as individual and mean fold change in proteins levels relative to siNT-transfected cells.

**Suppl. Figure 3: Clusterin overexpression in cardiomyocytes.** NCM were transfected by empty vector (pCMV6) or vector overexpressing the secreted isoform of clusterin (pCMV6 Clu) for 48h before MG132 treatment. Representative western blots (**A**) and quantification (**B**) of intracellular CLU protein levels (precursor and mature forms) and its secreted form in the 4 conditions (n=6/group). * indicates the nuclear isoform of clusterin. Sarcomeric actin (S-actin) was used to normalize intracellular proteins levels and data are expressed as individual and mean fold change in proteins levels relative to pCMV6-CLU transfected cells. Statistical significance was determined by Wilcoxon-Mann Whitney test. ***P*<0.01 *vs* non-stimulated condition or indicated values.

**Suppl. Figure 4: Secreted Clusterin decreased apoptosis induced by MG132 in rat cardiomyoblasts H9c2.** Rat cardiomyoblasts H9c2 stably transfected by the empty vector (pCMV6) or overexpressed the secreted isoform of clusterin (pCMV6-CLU) were treated or not with MG132 for 18h. **A:** Representative western blots and quantification of ubiquitinated proteins levels in the 4 conditions (n=9/group). . . Data are expressed as individual and mean fold change in proteins levels relative to pCMV6-CLU transfected cells. **B:** Representative western blots and quantification of cleaved caspase-3 proteins levels in the 4 conditions (n=9/group). Data are expressed as individual and mean fold change in proteins levels relative to MG132-treated pCMV6. **C:** Representative western blots and quantification of intracellular levels of clusterin (precursor, mature forms) and its secreted form in the 4 conditions (n=9/group). Data are expressed as individual and mean fold change in proteins levels relative to pCMV6-CLU transfected cells. β-actin was used to normalize intracellular proteins levels. Statistical significance was determined by Wilcoxon-Mann Whitney test. ***P*<0.01, ****P*<0.001 *vs* non-stimulated condition or indicated values*.*
