# Supplementary material for: Increased clusterin levels after myocardial infarction is due to a defect in protein degradation systems activity
Source: Cell Death Dis. 2019 Aug 13;10(8):608. doi: 10.1038/s41419-019-1857-x (PMC6691115; doi:10.1038/s41419-019-1857-x)
Supplement: Supplementary file 2 — Supplemental table 1 [file 41419_2019_1857_MOESM2_ESM.docx]

**Supplemental Table 1:** List and dilution of antibodies used for western blot analysis

| **Saturation**  **(before antibody incubation)** | **Primary antibodies** | **Incubation buffer** | **Sample :Dilution** | **Secondary antibodies** | **Dilution** |
| --- | --- | --- | --- | --- | --- |
| TBST-0.1% + 5% Milk | Clusterin  (sc-6419, Santa Cruz) | TBST-0.1% + 5% Milk | NCM lysate: 1/1000  NCM media: 1/3000  H9c2 Lysate: 1/250  H9c2 media: 1/250 | Anti-goat antibody  (sc-2354, Santa Cruz)  Or Anti-goat antibody  (sc-2020, Santa Cruz) | 1/5000  TBST-0.1% + 5% Milk  1/10000  TBST-0.1% + 5% Milk |
| TBST-0.1% + 5% Milk | S-Actin  (mo74, Dako) | TBST-0.1% + 5% Milk | 1/2500 | Anti-mouse antibody  (NA931, GE Healthcare) | 1/5000  TBST-0.1% + 5% Milk |
| TBST-0.1% + 5% Milk | β-Actin  (A5316, Sigma-Aldrich) | TBST-0.1% + 5% Milk | 1/20000 | Anti-mouse antibody  (NA931, GE Healthcare) | 1/20000  TBST-0.1% + 5% Milk |
| TBST-0.1% + 5% Milk | P62  (610498, BD Transduction Laboratories) | TBST-0.1% + 5% Milk | 1/2000 | Anti-mouse antibody  (NA931, GE Healthcare) | 1/5000  TBST-0.1% + 5% Milk |
| TBST-0.1% + 5% Milk | Bcl-2  (sc-493, Santa Cruz) | TBST-0.1% + 5% Milk | 1/1000 | Anti-mouse antibody  (NA931, GE Healthcare) |  |
| TBST-0.1% + 5% Milk | GAPDH  (sc-365062, Santa Cruz) | TBST-0.1% + 5% Milk | 1/10000 | Anti-mouse antibody  (NA931, GE Healthcare) | 1/20000  TBST-0.1% + 5% Milk |
| TBST-0.1% + 5% Milk | Beclin  (#3738, Cell Signalling) | TBST-0.1% + 5% BSA | 1/2000 | Anti-rabbit antibody  (NA934V, GE Healthcare) | 1/5000  TBST-0.1% + 5% Milk |
| TBST-0.1% + 5% Milk | LC3B  (#2775, Cell Signalling) | TBST-0.1% + 5% BSA | 1/1000 | Anti-rabbit antibody  (NA934V, GE Healthcare) |  |
| TBST-0.1% + 5% Milk | Cleaved caspase-3  (#9664, Cell Signalling) | TBST-0.1% + 5% BSA | 1/500 | Anti-rabbit antibody  (NA934V, GE Healthcare) |  |
| TBST-0.1% + 5% BSA | Mono-and-poly-ubiquitin  (BML-PW8810, Enzo Life Sciences) | TBST-0.1% + 5% BSA | 1/5000 | Anti-mouse antibody  (NA931, GE Healthcare) | 1/5000  TBST-0.1% + 5% BSA |
