# Supplementary figures and images for: Increased clusterin levels after myocardial infarction is due to a defect in protein degradation systems activity
Source: Cell Death Dis. 2019 Aug 13;10(8):608. doi: 10.1038/s41419-019-1857-x (PMC6691115; doi:10.1038/s41419-019-1857-x)

**A**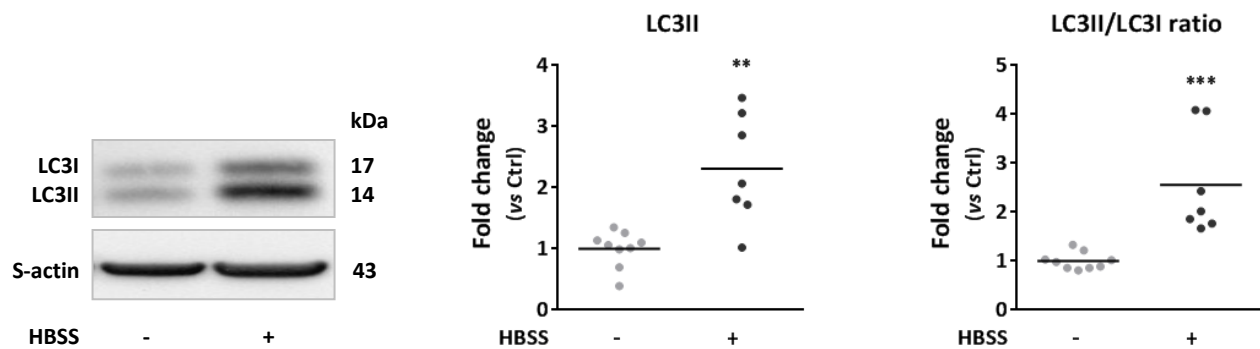**B**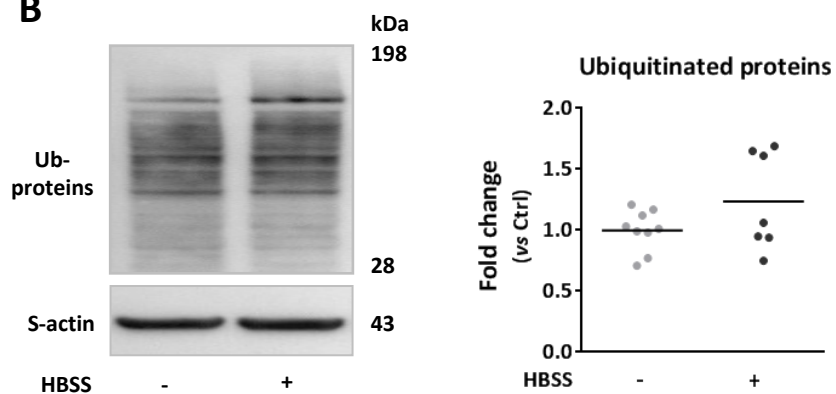**C**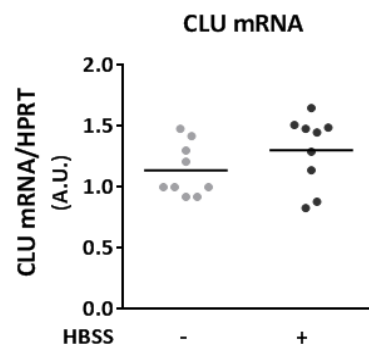**D**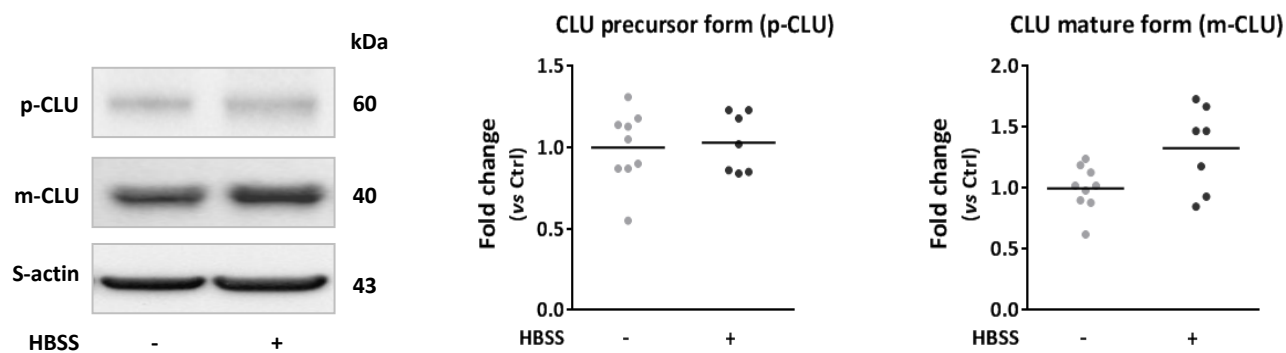

Supplement: Supplementary file 3 — Supplemental figure 1 [file 41419_2019_1857_MOESM3_ESM.pdf]

**A**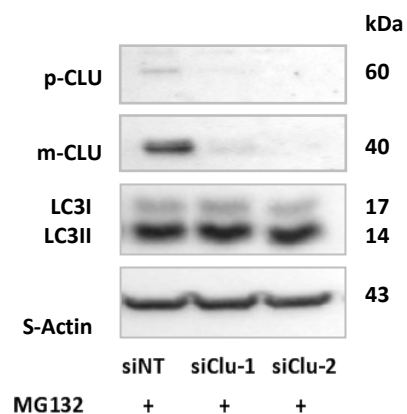**B**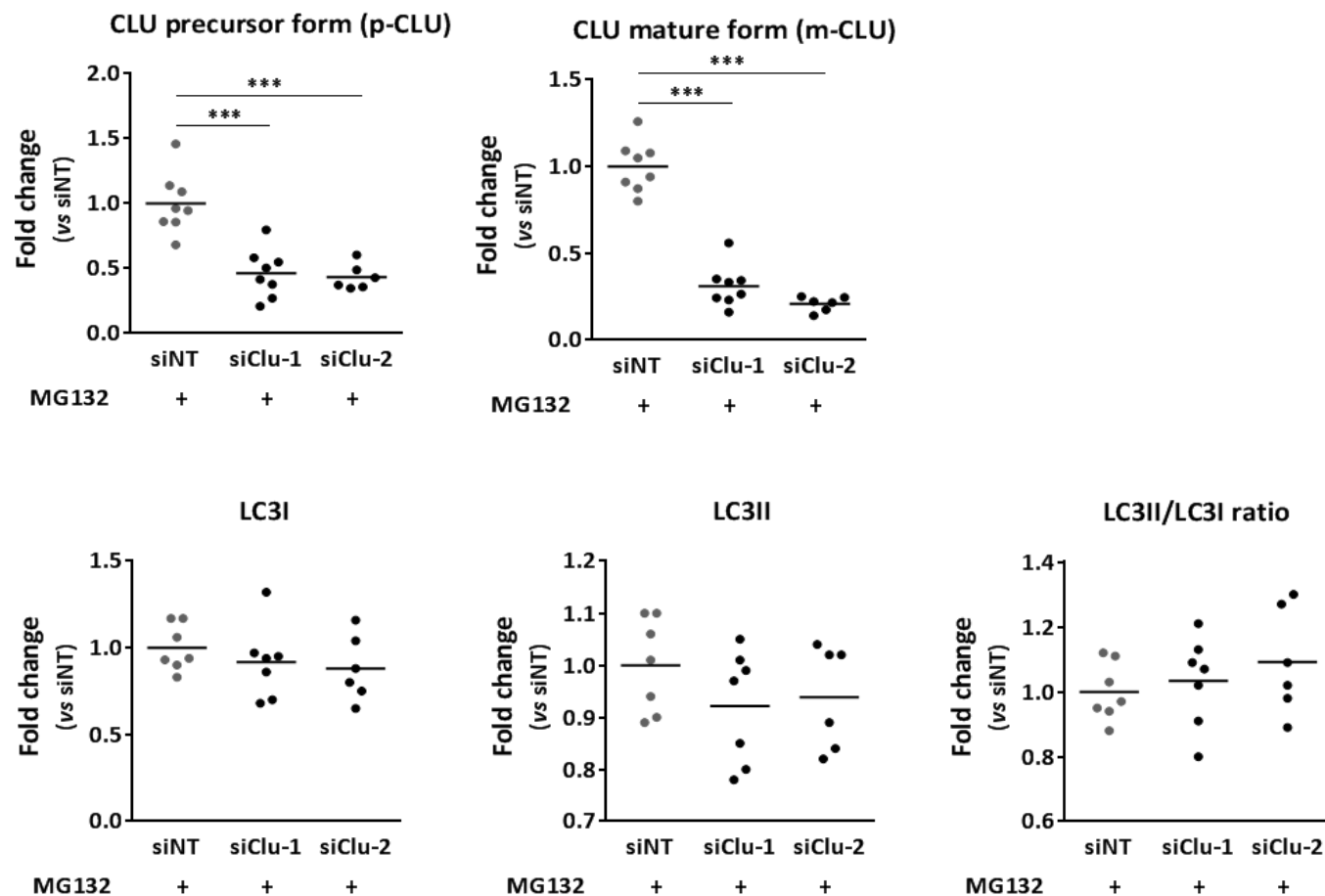

Supplement: Supplementary file 4 — Supplemental figure 2 [file 41419_2019_1857_MOESM4_ESM.pdf]

**A**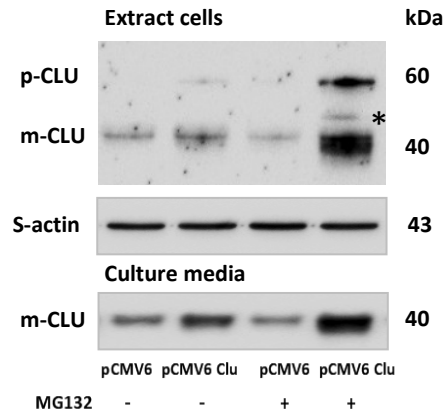**B**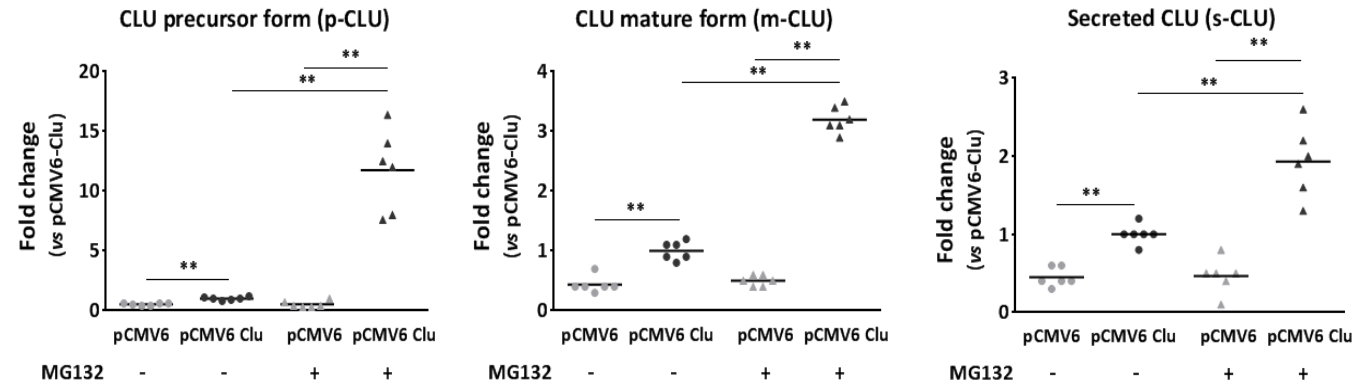

Supplement: Supplementary file 5 — Supplemental figure 3 [file 41419_2019_1857_MOESM5_ESM.pdf]

A

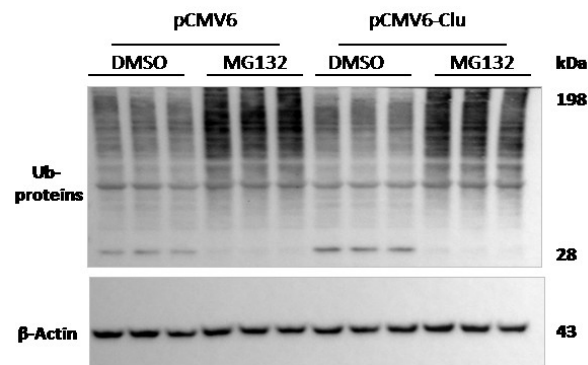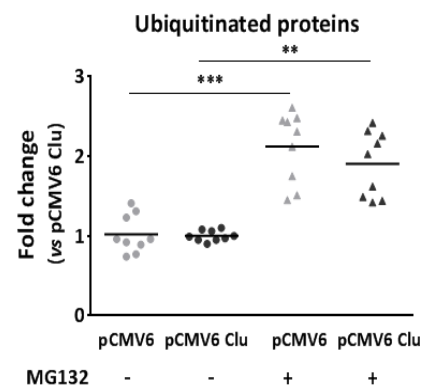

B

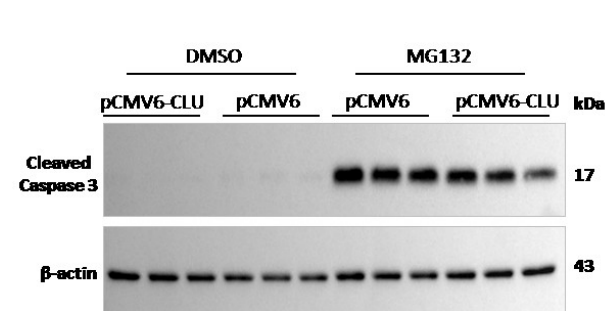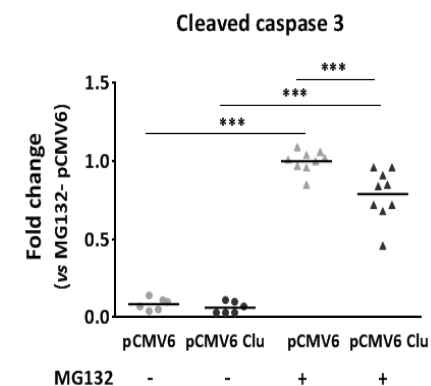

C

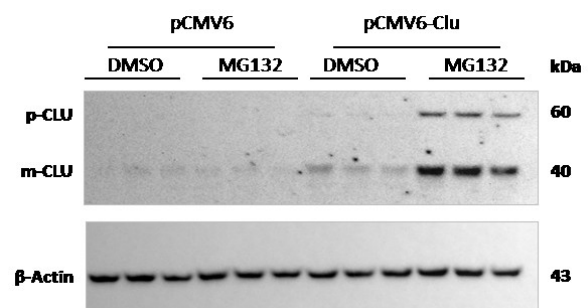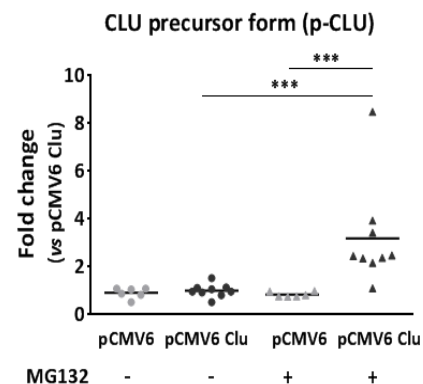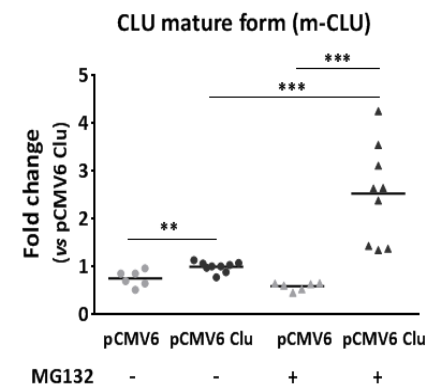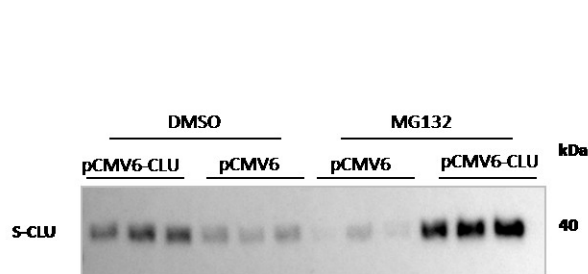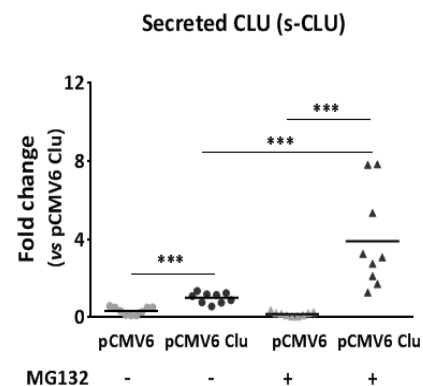

Supplement: Supplementary file 6 — Supplemental figure 4 [file 41419_2019_1857_MOESM6_ESM.pdf]
